# Panel‐based genetic testing for inherited retinal disease screening 176 genes

**DOI:** 10.1002/mgg3.1663

**Published:** 2021-03-22

**Authors:** Leo H. N. Sheck, Simona D. Esposti, Omar A. Mahroo, Gavin Arno, Nikolas Pontikos, Genevieve Wright, Andrew R. Webster, Kamron N. Khan, Michel Michaelides

**Affiliations:** ^1^ Moorfields Eye Hospital NHS Foundation Trust London UK; ^2^ Auckland District Health Board Auckland New Zealand; ^3^ UCL Institute of Ophthalmology University College London London UK; ^4^ St. James’s University Hospital Leeds UK

## Abstract

**Background:**

This case series reports the performance of a next‐generation sequencing (NGS) panel of 176 retinal genes (NGS 176) in patients with inherited retinal disease (IRD).

**Methods:**

Subjects are patients who underwent genetic testing between 1 August 2016 and 1 January 2018 at Moorfields Eye Hospital, London, UK. Panel‐based genetic testing was performed unless a specific gene (e.g., *RS1*) or small group of genes (e.g., *ABCA4*, *PRPH2*) were suspected. If a novel variant was identified, a further comment on their predicted pathogenicity and evolutionary conservation was offered and segregation studies performed. The main outcome measure is the likelihood of obtaining a genetic diagnosis using NGS 176.

**Results:**

488 patients were included. A molecular diagnosis was obtained for 59.4% of patients. Younger patients were more likely to receive a molecular diagnosis; with 92% of children under the age of 6 years receiving a conclusive result. There was a change in their initially assigned inheritance pattern in 8.4% of patients following genetic testing. Selected IRD diagnoses (e.g., achromatopsia, congenital stationary night blindness) were associated with high diagnostic yields.

**Conclusion:**

This study confirms that NGS 176 is a useful first‐tier genetic test for most IRD patients. Age and initial clinical diagnosis were strongly associated with diagnostic yield.

## Graphical abstract

A retrospective study of 488 patients with inherited retinal dystrophy confirms that NGS 176 is a useful first tier genetic test, achieving a molecular diagnosis in 59.4% of those tested. Age and initial clinical diagnosis were strongly associated with diagnostic yield.

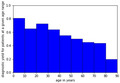

## 1 INTRODUCTION

Inherited retinal diseases (IRD) are a genetically heterogeneous group of conditions, the majority of which are currently untreatable. In England and Wales, IRD have now overtaken diabetic retinopathy as the most prevalent cause of sight impairment registration in working‐age adults, and are the second most common cause of sight loss in childhood (Liew et al., 2014; Rahi & Cable, 2003).

Managing patients with rare diseases requires specific expertise, which often includes access to diagnostic genetic testing, a field that has rapidly evolved over the past decade. While a range of techniques is now available, the optimal method of testing is yet to be established. For IRDs with a strong genotype‐to‐phenotype correlation, Sanger sequencing of single genes (e.g., *PAX6*, *RS1*, *CHM*, *KCNV2*, recessive *NR2E3*) or even single amplicons (e.g., *EFEMP1*:c.1033C>T, p.(Arg345Trp); *C1QTNF5*:c.489C>A, p.(Ser163Arg); m.3243a>g) may be the most cost‐effective approach. For others, such as patients presenting with the classical features of rod‐cone dystrophy (retinitis pigmentosa), pathogenic variants in any one of over 100 different genes could be disease‐causing, and consequently, a less focussed approach may be advantageous. Such techniques may involve either targeted capture and then resequencing of genomic regions known to harbor IRD genes, or more recently, whole exome or whole genome sequencing, typically followed by the bioinformatic masking of non‐IRD causing genes (to minimize the detection of “incidental findings”). Whichever technique is used, once sequencing of IRD genes has taken place, interpreting the significance of detected variants is the next challenge, often necessitating a multidisciplinary team approach.

Despite these challenges, genetic testing is highly valued by patients and clinicians, primarily as it helps to provide an accurate diagnosis, and consequently, inform prognosis (Broadgate et al., 2017). Clinical management may also be directly influenced (renal disease, SCA7, CLN3) (Ellingford et al., 2015; Hamel, 2007), and in a minority of cases treatment initiated (e.g., adult Refsum disease and gyrate atrophy) (Orphanet: Gyrate atrophy of choroid & retina; Orphanet: Refsum disease), which, for recessive *RPE65*‐associated IRD, may now involve gene‐replacement therapy (Office of the Commissioner Press Announcements; Russell et al., 2017). Achieving a confirmed molecular diagnosis opens the possibility to be involved in the treatment trial, and so far there are active gene therapy trials on *RPGR, CHM, MERTK, RS1, and PDE6B* (Home ‐ ClinicalTrials.gov). Apart from gene therapy, novel pharmacotherapy (e.g., C20‐deuterated vitamin A for *ABCA4* disease, neuroprotection, optogenetic therapy, stem cell therapy, and retinal prosthesis are all under various stages of development, and most of these would require a clear molecular diagnosis as a prerequisite for inclusion in clinical trials (Scholl et al., 2016). Last, a molecular diagnosis enables accurate counseling regarding the disease recurrence risk, as well as access to pre‐natal interventions (Brezina et al., 2012).

In previous work, we have described our experience using a 105 gene panel (Khan et al., 2017), which provided a conclusive molecular diagnosis for 39% of our patients with IRD. The present work now reports our experience of genetic testing, in a similar cohort of patients, using the next iteration of this test, which now screens 176 genes (NGS 176).

## 2 SUBJECTS AND METHODS

The methods in this study are similar to our previous publication (Khan et al., 2017). Briefly, the results of all NGS 176 tests received between 1 August 2016 and 1 January 2018 were retrospectively reviewed. All patients had been diagnosed with an IRD by one of the three experienced clinicians (A. T. M., A. R. W., M. M.) at Moorfields Eye Hospital, London, UK. Where a specific gene (e.g., *RS1*), or small group of genes were suspected (e.g., *ABCA4*, *PRPH2*), patients were not subjected to the full 176 gene screen, and a molecular diagnosis was obtained by focused exome sequencing of 10 genes known to cause macular dystrophy (Stargardt/Macular dystrophy panel v3, Casey Eye Institute Molecular Diagnostics Laboratory). Similarly, when X‐linked rod‐cone dystrophy or choroideremia was suspected, single‐gene sequencing was performed (ORF15 of *RPGR*, exons 114 *RPGR*, *RP2*, and *CHM*, respectively) (National Genetics Reference Laboratory, Manchester Center for Genomic Medicine). Patients with albinism, isolated foveal hypoplasia, and inherited optic neuropathies were typically investigated using alternative pathways. However, retinal dystrophies occurring as a part of syndromic diagnosis were included.

The following patient demographics were extracted from the hospital electronic medical records (OpenEyes): age at the time of genetic testing, ethnicity, clinical diagnosis, and suspected mode of inheritance (when commented upon).

### 2.1 Genetic testing

A custom‐designed (Retinal dystrophy v3) Sure Select Target Enrichment Kit (Agilent Technologies, Santa Clara, CA) was used for targeted enrichment of 176 genes and immediate splice sites ±5 bases known to be mutated in patients with isolated and syndromic retinal disease (Manchester Centre for Genomic Medicine). The samples were sequenced using a HiSeq 2500 (Illumina) according to the manufacturer's protocols. Sequence data were aligned to the hg19 human genome using BWA‐MEM v0.6.2 and abra v0.96. Variant calling was completed using Genome Analysis Tool Kit (GATK‐lite v2.0.39) (SNVs and indels), Pindel v0.2.4t (large indels), and DECoN v1.0.1 (copy number variation). Using this approach, sequencing data from 99.5% of all coding exons from 176 genes are obtained, with a minimum accepted read depth of 50X. The testing laboratory (Manchester Center for Genomic Medicine, Manchester UK) then issues a clinical report detailing variants thought to account for the disease. If a novel variant is identified, a further comment as to the predicted pathogenicity and evolutionary conservation was also offered and segregation studies performed.

### 2.2 Statistical analysis

Statistical testing was performed using Python (Welcome to Python.org), ResearchPy (Bryant, 2020), and Statsmodels (StatsModels: Statistics in Python — statsmodels 0.9.0 documentation). The chance of achieving a confirmed genetic diagnosis was assessed by univariate analysis. Continuous variables were analyzed using Student's *T* test, and categorical variables were analyzed using a Chi‐squared test. Logistic regression was used to model the interactions between the independent variables (age, sex, initial clinical diagnosis, suspected inheritance pattern, ethnicity) and the outcome of achieving a confirmed genetic diagnosis. The Wald test was used to assess the significance of the interaction at the overall variable level. If this was found to be statistically significant, further analysis was performed for individual categories within the variable to determine the odds ratio, and p‐values returned within the logistic regression model. A result was deemed statistically significant if its p‐value was less than 0.05.

## 3 RESULTS

Genetic results for 488 patients were received between 1 August 2016 and 1 January 2018. The mean age of the tested patients was 38 years, with a range of 0–88 years. Detailed demographic data are presented in Table 1.

**TABLE 1:** Demographic data and result of genetic testing using NGS 176 panel

Number of patients	488
Age (mean, SD, range)	38, 20, 0–88
Percentage male	53.90%
Ethnicity (*n*)	
Not stated	199
European	135
Non‐European	154
Result of testing (*n*) percentage	
Confirmed molecular diagnosis	(290) 59.4%
Inconclusive	(45) 9.2%
No pathogenic mutation found	(153) 31.4%

The results of genetic testing were categorized as follows: (a) testing established a conclusive molecular diagnosis in regard to the genotype of the patient (all/sufficient likely disease‐causing alleles were identified), (b) testing was inconclusive in regard to the genotype of the patient (not all disease‐causing alleles were identified/only variants of uncertain significance were detected), and (c) no pathogenic variants thought to account for the phenotype were identified in the patient concerned. Table 2 shows the findings after NGS 176 in these patients. Overall, a conclusive molecular diagnosis was possible for 59.4% of the study cohort (Table 1).

**TABLE 2:** Clinical diagnosis of patients in this study and the associated NGS 176 gene panel diagnostic yield

Diagnosis	Number	Age in years (mean ±standard deviation)	diagnostic yield (% confirmed molecular diagnosis)
Rod‐cone dystrophy	297	41 ± 18	60.9%
Young (<10 years)	11		81.8%
Juvenile (10–20 years)	42		64.3%
Adult (20–40 years)	82		68.3%
Late adult (>40 years)	162		56.8%
Retinal dystrophy	74	37 ± 22	48.7%
Cone‐rod dystrophy	30	41 ± 20	56.7%
Macula dystrophy	27	42 ± 17	37.0%
Cone dystrophy	25	25 ± 18	80.0%
Vitreoretinopathy	19	27 ± 20	36.8%
Congenital stationary night blindness	16	15 ± 21	93.8%

Retinal dystrophy ‐ cases where patients did not fit into any existing clinical classification but had bilateral, symmetrical loss of vision thought to be due to a Mendelian disease. Vitreoretinopathy – this include x‐linked retinoschisis, familial exudative vitreoretinopathy, and other forms of vitreoretinopathy. Cone dystrophy – this includes achromatopsia and blue cone monochromacy. Rod‐cone and cone‐rod dystrophies were differentiated based on their initial, predominant symptom (either abnormal scotopic or photopic function).

**FIGURE 1:**
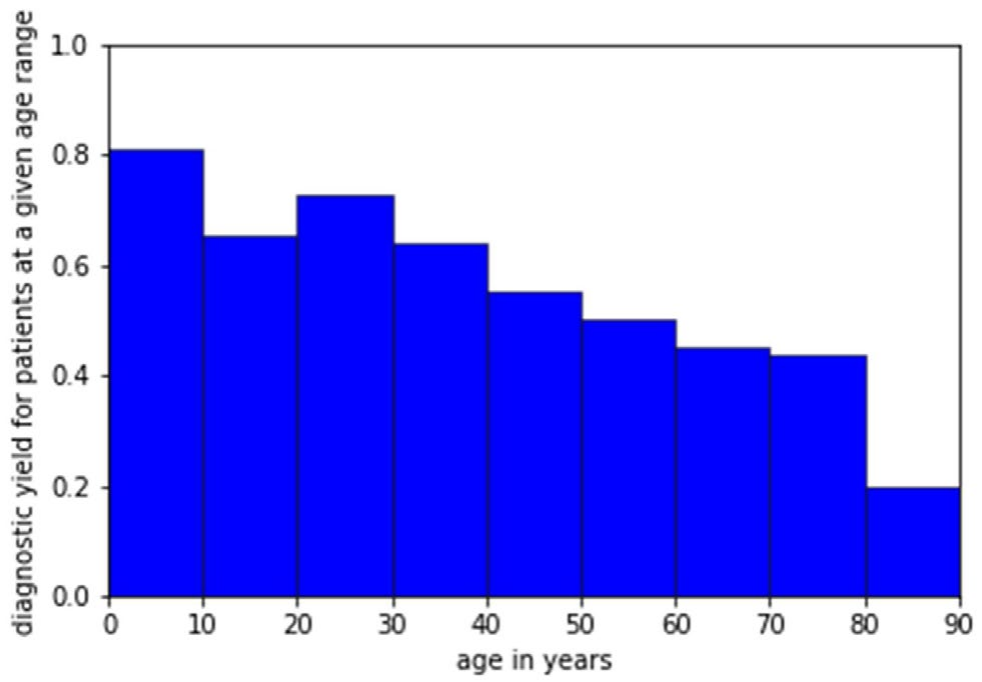
Diagnostic yield for patients within a given age range within this study

We then explored the influence of patient age at the time of testing on the likelihood of obtaining a conclusive molecular diagnosis. We defined diagnostic yield as the proportion of patients with a conclusive molecular diagnosis in that category. Figure 1 plots the diagnostic yield of the NGS 176 panel for patients at any given age. For patients aged 6 or less, a positive result was obtained for 91.8%. This reduced to 72.0% for all patients under the age of 35. The diagnostic yield continued to decline with increasing age. These results are highly statistically significant on both univariate (*p* = 1.22E‐07) and multivariate analysis (*p* = 1.29E‐06) (Tables 3 and 4).

**TABLE 3:** Univariate analysis on the correlation between diagnostic yield, and age, sex, ethnicity, suspected inheritance pattern, and clinical diagnosis

Age						
NGS result	*N*	Mean	SD	SE	95% CI	
*n*	198	43.9	19.7	1.4	41.2	46.7
*y*	290	34.1	19.8	1.2	31.9	36.4
*p* value	1.22E‐07*					

Inheritance				
	AD	AR	Unclear	x‐linked
Diagnostic yield (%)	70	57	58	76
Total number	60	375	36	17
Pearson Chi‐square	5.71			
*p* value	0.13			
Cramer's V	0.11			

Sex		
	*m*	*f*
Diagnostic yield (%)	56.3	63.1
Total number	263	225
Pearson Chi‐square	2.35	
*p* value	0.13	
Cramer's V	0.07	

Diagnosis							
	Cone dystrophy	Cone‐rod dystrophy	Macula dystrophy	Congenital stationary night blindness	Retinal dystrophy	Rod‐cone dystrophy	Vitreoretinopathy
Diagnostic yield (%)	80.0	60.0	37.0	93.8	48.7	62.0	36.8
Total number	25	30	27	16	74	297	19
Pearson Chi‐square	26.19						
*p* value	0.0002*						
Cramer's V	0.23						

Ethnicity			
	White	Not stated	Other
Diagnostic yield (%)	55.6	65.3	55.2
Total number	135	199	154
Pearson Chi‐square	4.86		
*p* value	0.088		
Cramer's V	0.10		

AD, autosomal dominant; AR, autosomal recessive; CI, confidence interval; f, female; m, male; N, number; SD, standard deviation; SE, standard error.

* Denotes a statistically significant result.

**TABLE 4:** Multivariate analysis of the correlation between diagnostic yield, and age, sex, ethnicity, suspected inheritance pattern and clinical diagnosis

Wald test	Statistic	*p* value
Sex	2.90	0.089
Diagnosis	20.49	0.0023*
Inheritance	5.49	0.14
Ethnicity	1.78	0.41
Age	23.45	1.29E−06*

	odds ratio	*p* value
Age	0.97	1.29E‐06*
Diagnosis (odds ratio as compared to rod‐cone dystrophy)		
Cone dystrophy	1.74	0.30
Cone‐rod dystrophy	0.95	0.90
Macula dystrophy	0.35	0.016*
Congenital stationary night blindness	5.43	0.13
Retinal dystrophy	0.51	0.022*
Vitreoretinopathy	0.25	0.0077*

Wald test is used to assess statistical significance at the level of the independent variables, and the result of the logistic regression is given for those independent variables reaching statistical significance in the Wald test.

* Denotes statistically significant result.

We then investigated whether the prevalence of null alleles (variants that are predicted to either directly or indirectly result in a premature termination codon) differed according to patient age at testing. The results are presented in Table 5; we did not detect a difference in age between patients carrying (single/double) null and missense only alleles.

**TABLE 5:** comparing the age of patients with the number of nonsense mutations per patient showing no statistically significant relationship

Number of nonsense mutations	Number of patients	Mean age	Standard deviation	95% confidence interval	
Autosomal recessive					
0	87	31.5	20.3	27.2	35.8
1	49	34.6	18.5	29.4	39.9
2	62	33.1	18.6	28.5	37.8
Autosomal dominant or x‐linked					
0	45	40.5	19.6	34.7	46.3
1	46	33.9	21.4	27.6	40.1

We then examined whether the diagnostic yield varied according to patients’ initial clinical diagnosis. Table 2 presents these data, and Tables 3 and 4 the corresponding statistical analysis. Patients diagnosed with congenital stationary night blindness (CSNB; based on normal retinal structure and abnormal electroretinography), were most likely to receive a definitive molecular diagnosis (93.8%), followed by patients diagnosed with a stationary or progressive cone disorder (80%). This high diagnostic yield in disorders of cone function is partly due to a 100% diagnostic yield for patients with achromatopsia. Individuals with either a rod‐cone or cone‐rod dystrophy had a similar diagnostic yield (56.7% and 60.9%). While patients diagnosed with a (not further classified) “retinal dystrophy” had less chance of receiving a molecular diagnosis (48.7%). In this cohort, only 37% of patients diagnosed with macular dystrophy received a conclusive genetic diagnosis. The relationship between initial clinical diagnosis and diagnostic yield was statistically significant in both univariate (*p* = 0.0002) and multivariate analysis (*p* = 0.0023). The specific diagnoses of macular dystrophy and undefined retinal dystrophy have a lower odds ratio in diagnostic yield comparing to rod‐cone dystrophy (macular dystrophy odds ratio 0.35, p‐value 0.016; undefined retinal dystrophy odds ratio 0.51, *p*‐value 0.022).

The influence of the mode of inheritance on diagnostic yield was also explored. These data are presented in Table 6. Autosomal dominant (AD) inheritance was proposed when multiple generations were affected, without a history of consanguinity. X‐chromosome linked (XL) disease was suggested when male family members were consistently more severely affected than females, with no male‐to‐male inheritance. Autosomal recessive (AR) disease was considered most likely in consanguineous or endogamous pedigrees, or in simplex cases when both parents were unaffected. In this cohort, patients suspected to have an AR disease appeared to be less likely to receive a conclusive molecular diagnosis compared to patients predicted to segregate an XL or AD condition (Table 7); although this did not reach statistical significance on both univariate and multivariate analyses. Molecular genetic testing corrected the suspected mode of inheritance in 8.4% of patients. Of particular relevance to genetic counseling, 20% of patients thought to have an XL condition had an alternative pattern of inheritance (13% AD, 6.7% AR), and for 9.1% of patients suspected to have AR disease this too was incorrect (6.7% cases were reassigned AD, 2.4% of cases were, in fact, XL, all these cases are *de novo* cases with both parents unaffected).

**TABLE 6:** suspected inheritance pattern based on the clinical data of patients in this study and the associated NGS 176 gene panel diagnostic yield

Suspected inheritance pattern	number	Age (mean, standard deviation)	Diagnostic yield (% confirmed molecular diagnosis)
Autosomal recessive	375	38 ± 20	56.0%
Autosomal dominant	60	44 ± 17	70.0%
x‐linked	15	29 ± 22	86.7%
not commented upon	36	33 ± 22	58.3%

**TABLE 7:** number (percentage within the category) of patients with a change between the clinically suspected inheritance pattern and the confirmed inheritance pattern following genetic testing

Suspected inheritance pattern	Confirmed inheritance pattern	n, (%)
Autosomal dominant	Autosomal recessive	3 (5.0%)
Autosomal dominant	x‐linked carrier	1 (1.7%)
Autosomal recessive or simplex	Autosomal dominant	24 (6.4%)
Autosomal recessive or simplex	x‐linked	10 (2.7%)
x‐linked	Autosomal dominant	2 (13%)
x‐linked	Autosomal recessive	1 (6.7%)
Total		41 (8.4%)

In this study, the most prevalent genetic cause of AR IRD were disease‐causing variants in *USH2A* (n = 37, 9.8% of all autosomal recessive or simplex cases). Variants in *RP1* were the most common cause of autosomal dominant disease (n = 19, 31.7%). Here, pathogenic variants in *CNGB3* were the most common cause of achromatopsia (ACHM), and variants in *CACNA1F* were the most prevalent cause of CSNB. Table S1 provides a more detailed analysis.

## 4 DISCUSSION

The use of phenotype‐based gene panel testing facilitates the rapid interpretation of targeted capture, whole‐exome, and whole‐genome sequencing (WGS) data, thus offering great clinical utility (Broadgate et al., 2017). The development of these panels is iterative and evolves based on contemporary knowledge. The present study analyses the performance of the latest molecular test (NGS 176) used in the UK National Health Service to screen for IRD, reporting a diagnostic yield of 59%. This out‐performed the previous gene screen from the same laboratory (NGS 105), which had a yield of 39% under similar testing conditions (Khan et al., 2017). As patients presenting with a clearly recognizable macular dystrophy phenotype (including those associated with variants in *ABCA4*, *PRPH2*, *RS1*, *BEST1*) and X‐linked rod‐cone dystrophy/Choroideremia (*RPGR*, *RP2*, *CHM*) were investigated by alternative pathways, the diagnostic yield of NGS176 for all patients with IRD is likely to be significantly higher than we report. Despite this, the yield of 59% compares favorably with other international IRD cohorts—57.6% (Stone et al in a tiered approach without exome / genome sequencing) and 50% (Ellingford et al using a 105 gene panel, similar to our previous study) (Ellingford et al., 2016; Stone et al., 2017).

Molecular testing, especially for genetically heterogeneous conditions such as IRD, may be expected to have a diagnostic yield of less than 100% for a number of reasons, which may be broadly categorized as follows: (a) the causative genetic locus may not be included in the testing panel (eg *DRAM2*, *RAX2*—both recently reported novel causes of IRD that were unknown at the time of NGS 176 panel design, or *CHM*: chrX: 85,220,593T>C (ENST00000357749.2; c.315–1536A>G), a recently identified pathogenic intronic variant in a gene that is included in the panel); (b) there is insufficient genetic data available after sequencing a known disease‐causing locus due to its inherent complexity (e.g., ORF15 of *RPGR*); or (c) current genetic analysis is not sophisticated enough to interpret the sequencing data accurately (mapping of sequence reads for the X‐chromosome Opsin array, detection of medium‐sized indels or complex structural variants, interpreting the clinical significance of detected exonic or intronic variants). A range of different techniques are being used to address each of these issues, one of which involves sequencing additional genetic loci. These now include both exonic and intronic regions, and some IRD panels now including up to 875 genes (Retinal Dystrophy Xpanded panel, GeneDx). Although “physical” gene panels are updated infrequently, primarily due to the time and cost involved, bioinformatic refreshing of “virtual” gene panels is far more efficient. This approach is becoming increasingly prevalent as the price of whole‐exome and whole‐genome sequencing falls. In addition, novel techniques, such as long‐read sequencing, now enable repetitive and GC‐rich genomic regions to be interrogated in an unbiased manner, facilitating the detection of hitherto elusive variants (Mizuguchi et al., 2019). Using these approaches, the uplift in diagnostic yield has been suggested to be in the range of 18–20% by one group (Stone et al., 2017), and 29% by another (Ellingford et al., 2016). Carss et al. report a more conservative detection rate of 55% in a cohort of 605 patients analyzed using WGS (Carss et al., 2017). As the cost of sequencing falls, other steps in the genetic testing pipeline replace financing as the next significant challenge to overcome; these include sample handling, quality control, bioinformatic analysis, report writing, segregation studies, and delivering accurate genetic counseling.

In this study, age was highly correlated with the likelihood of obtaining a genetic diagnosis. Although the age at which patients first developed symptoms could not be accurately ascertained, we recorded age at the time of genetic testing as a surrogate marker for this, accepting its inherent limitations. Genetic testing in children with IRD had a significantly higher success rate than when the same test was performed in older adults, an important consideration when performing pretest counseling. A number of explanations for this observation may exist. First, congenital or early‐onset IRD represent major departures from normal physiology, which we would expect to be mediated by a rare genetic variant of high impact. In contrast, conditions with an adult‐onset, suggest a process that is likely to have taken two or more decades to develop, and consequently result from less penetrant alleles. These variants may, therefore, be under less selective pressure, and so be more prevalent than those associated with childhood‐onset disease. This is perhaps best exemplified by disease‐causing variants in *ABCA4*, where those associated with childhood‐onset autosomal recessive Stargardt disease (STGD1) usually function as null alleles and are rare, while those associated with late‐onset STGD1 are often hypomorphic, have a greater prevalence in the general population, and so are less readily identifiable using current bioinformatics analysis (Fujinami et al., 2015). As a result, it is not uncommon for genetic testing to only identify one of the two disease‐causing *ABCA4* variants in cases of adult‐onset STGD1, a situation that rarely occurs in childhood‐onset cases (Fujinami et al., 2015). Second, the functional consequences of a variant are also likely to relate to their genomic position, with exonic, non‐synonymous variants predicted to be of larger effect size, overall, compared to variants in non‐protein‐coding regions; however, this is not always the case. Notable exceptions relevant to IRD include variants in the non‐coding region upstream to *PRDM13* associated with North Carolina Macular Dystrophy (Small et al., 2016) (OMIM 13650) and *CEP290*:c.2991+1655A>G, a prevalent cause of LCA in European patients (Sheck et al., 2018). Typically, exonic variants are easier to detect and their significance easier to interpret, and so current pipelines are optimized to detect these variants that may be of greater biological significance. In a pooled analysis we were not able to detect a difference in age between patients with one/multiple null alleles versus those with only missense variants. Reasons for this may include (a) the effect of missense alleles being hard to discern and many, may in fact function as null alleles; (b) significant genotype‐specific data being lost in a pooled analysis (e.g., for *CACNA1F*); and (c) the exclusion of specific genotypes that were investigated using alternative pathways (e.g., *ABCA4*). Last, it is also possible that a number of patients diagnosed with late‐onset IRD may in fact have an acquired disorder mimicking IRD. Known phenocopies of IRD include some forms of inflammatory retinal disease (uveitis), toxic retinopathies (e.g., that associated with hydroxychloroquine or pentosan polysulfate use), and autoimmune retinopathies (Gregory‐Evans et al., 2018). If this cohort of typically older patients were erroneously offered genetic testing, a lower yield would be expected.

In addition to age, the clinical diagnosis was found to be significantly associated with diagnostic yield after multivariate analysis (Tables 3 and 4). The most genetically homogeneous IRD phenotypes, ACHM and CSNB, associated with 6 and 10 genes, respectively, had the highest diagnostic yield, 100% and 94%, while more heterogeneous subgroups (rod‐cone dystrophy associated with >100 genes) had much lower yields (61% overall). Genetic testing was least informative for less well‐classified cases (here termed retinal dystrophy and vitreoretinopathy) (Table 6), suggesting that either the clinical diagnosis of IRD may have been questionable, that the disorder may not be inherited as a Mendelian trait, or, if monogenic, that the causative variant resides in a genomic locus that is currently poorly understood or not represented on the 176 gene panel. Within the largest IRD subgroup, those with rod‐cone dystrophy, the youngest patients were more likely to receive a conclusive result from genetic testing (82%) when compared with the oldest patients tested (57%). It is likely that the low diagnostic yield for patients with macular dystrophy is a result of the genetic testing algorithm used here, as patients with phenotypic clues as to their genotype (vitelliform lesion suggestive of *BEST1*‐associated disease, schisis suggestive of *RS1*, flecks indicative of *ABCA4* or *PRPH2*) were investigated by another route.

Over time, the preferred technique for genetic testing has evolved, and in the United Kingdom, at least for patients with rare disease and cancer, the future promises increased access to WGS (The 100,000 Genomes Project). However, short‐term panel testing will remain the principal option for IRD screening in the United Kingdom—partly due to cost considerations but also due to the high diagnostic yield illustrated in this report. While WGS will mitigate against some of the technical causes for loss in diagnostic yield observed in this study, it brings with it its own significant challenges (Stone et al., 2017). A phenotype‐based gene panel approach to interpreting WGS data is likely to offer great clinical utility, with limited off‐target effects. Sharing detailed knowledge of rare variants will become increasingly important, and depositing these data in publically accessible repositories will facilitate patient care worldwide. This work serves to highlights how effective current genetic testing in the NHS is, and how it can be used to care for 1 in 2–3,000 patients with IRD (Hartong et al., 2006).

## 5 CONFLICT OFINTERESTS

The authors declare no competing interests.

## AUTHOR’S CONTRIBUTIONS

LS, KK, and MM conceived and designed the analysis. LS and SE collected the data. OM, GA, GW, and AW contributed the data. LS and NP performed the bioinformatics analysis. LS performed the statistical analysis. LS and KK wrote the manuscript. KK, OM, MM, and AW critically appraised and revised the manuscript. LS, KK, and MM gave the final approval for the work to be published. All authors have participated sufficiently in the work to take public responsibility for appropriate portions of the content and agreed to be accountable for all aspects of the work.

## Supporting information

Table S1

## Data Availability

The data that support the findings of this study are available on request from the corresponding author. The data are not publicly available due to privacy or ethical restrictions.
